# Urbanisation, urbanicity, and health: a systematic review of the reliability and validity of urbanicity scales

**DOI:** 10.1186/1471-2458-13-513

**Published:** 2013-05-28

**Authors:** Sheila Cyril, John C Oldroyd, Andre Renzaho

**Affiliations:** 1 Global Health and Society Unit, School of Public Health and Preventive Medicine, Monash University, 99 Commercial Road, Melbourne, VIC 3004, Australia; 2 Migration, Social Disadvantage, and Health Programs, Global Health and Society Unit, Monash University, 99 Commercial Road, Melbourne, VIC 3004, Australia; 3 Centre for International Health Burnet Institute, School of Public Health and Preventive Medicine, Level 3, 89 Commercial Road, Melbourne, VIC, 3004, Australia

**Keywords:** Urbanisation, Urbanicity scale, Measurement, Reliability, Validity, Health

## Abstract

**Background:**

Despite a plethora of studies examining the effect of increased urbanisation on health, no single study has systematically examined the measurement properties of scales used to measure urbanicity. It is critical to distinguish findings from studies that use surrogate measures of urbanicity (e.g. population density) from those that use measures rigorously tested for reliability and validity. The purpose of this study was to assess the measurement reliability and validity of the available urbanicity scales and identify areas where more research is needed to facilitate the development of a standardised measure of urbanicity.

**Methods:**

Databases searched were MEDLINE with Full Text, CINAHL with Full Text, and PsycINFO (EBSCOhost) as well as Embase (Ovid) covering the period from January 1970 to April 2012. Studies included in this systematic review were those that focused on the development of an urbanicity scale with clearly defined items or the adoption of an existing scale, included at least one outcome measure related to health, published in peer-reviewed journals, the full text was available in English and tested for validity and reliability.

**Results:**

Eleven studies met our inclusion criteria which were conducted in Sri Lanka, Austria, China, Nigeria, India and Philippines. They ranged in size from 3327 to 33,404 participants. The number of scale items ranged from 7 to 12 items in 5 studies. One study measured urban area socioeconomic disadvantage instead of urbanicity. The emerging evidence is that increased urbanisation is associated with deleterious health outcomes. It is possible that increased urbanisation is also associated with access and utilisation of health services. However, urbanicity measures differed across studies, and the reliability and validity properties of the used scales were not well established.

**Conclusion:**

There is an urgent need for studies to standardise measures of urbanicity. Longitudinal cohort studies to confirm the relationship between increased urbanisation and health outcomes are urgently needed.

## Background

Over the past few decades, there has been a rapid urbanisation of the world’s population. The United Nations’ Department of Economic and Social Affairs indicate that in 2007, 74% of the population in more developed regions lived in urban areas, compared with just 44% in less developed regions [1]. It projects that urbanisation will continue to rise in both developed and low- and middle-income countries (LMICs) and 70% of the world’s population (86% for developed countries and 67% for LMICs) will be living in urban areas by 2050, but the pace of urbanisation will be greater in LMICs than developed countries [1].

However, defining urbanisation is difficult and there has been a plethora of definitions put forward by various researchers [2-4]. The most commonly used definition conceptualises *urbanisation* as “*change in size, density, and heterogeneity of cities*” p. S1 [5]. This could be a result of rural to urban migration or a natural population increase due to a decrease in death rates while birth rates remain high [1]. Urbanisation brings with it both positive and negative dimensions [6]. Positive elements include internal commerce and foreign trade, financial services and economic growth, growth of modern production and industry, education, and other government services [6]. Negative factors that affect health include increased car ownership which results in traffic congestion and air pollution, lack of green areas, creation of slums (illegal real estate and development), sewerage pollution, strain on existing urban infrastructure (e.g. transport, housing and health care) due to a rapid increase in population density, and social problems such as socio-economic inequalities (poverty), prostitution, and crime [6,7].

In contrast, the term *urbanicity* refers to “*the impact of living in urban areas at a given time*” p. S1 [5]. It is static, that is, it refers to the urban conditions at any given point in time rather than a ‘process’ of a city’s changing characteristics. It refers to “*the presence of conditions that are particular to urban areas or present to a much greater extent than in nonurban areas*” (e.g. industrial pollution, congestion, motor vehicle accidents) [5]. The effects of urbanicity refers to the contrast between an already existing city and its surrounding area (e.g. in a developed country the contrast between a city and the suburbs and rural areas around it). Hence, urbanicity is complementary to urbanization and both dimensions shape health. An urbanicity index provides a simple quick method of measuring the degree of urbanisation and examining the effects of urban living on health [5].

The United Nations has recognised that there are many adverse health outcomes associated with greater urbanisation, including increased communicable and non-communicable diseases [8]. Studies have consistently shown that high urbanisation is associated with an increased risk of chronic disease such as higher prevalence of type 2 diabetes, hypertension and the metabolic syndrome [9-11]. Similarly, increased urbanisation has been found to be associated with an array of risk factors. For example, increased urbanisation is associated with noise pollution which leads to hearing impairment, sleep disturbances, stress-related disorders and cognitive impairments [12] while increased urban exposure to air pollution leads to development of asthma in children and adolescents and the exacerbation of asthmatic symptoms in adults [13-15].

A study by Dahly and Adair found a positive, linear relationship between urbanicity and total calorie intake and the percentage of dietary fat [3]. In contrast, they found an inverse relationship between urbanicity and breastfeeding behaviours (e.g. exclusively breastfeeding and breast feeding duration). A shift in dietary, physical activity and obesity patterns central to nutrition transition is seen commonly in LMICs urban populations [16]. Other adverse effects on health and behaviour associated with increased urbanicity include increased HbA1c levels, hypertension, and overweight/obesity [17,18]; increased fat intake, decreased physical activity [19]; smoking [20]; increased traffic flow [21]; overcrowding and poor hygiene and sanitation [3]; high levels of air pollution [22]; reduction in occupational activity [23]; risky sexual behaviour [24,25]; and increased tobacco, alcohol and drug use, increased levels of psychological stressors, higher crime rate, homicide, suicide and mental health disorders as well as higher level of injury including motor vehicle accidents [3,5,25,26].

Despite a plethora of studies examining the effect of increased urbanisation on health, no single study has systematically examined the reliability and validity of scales used to measure urbanicity. Accurately measuring urbanicity and evaluating the robustness of the existing scales is of prime importance in developing effective strategies to combat social and health issues associated with increased urbanisation. It is critical to distinguish findings from studies that use surrogate measures of urbanicity (e.g. population density) from those that use reliable and valid measures. Therefore, the purpose of this study was to assess the properties of the available urbanicity scales (reliability and validity) and identify areas where more research is needed to facilitate the development of a standardised measure of urbanicity.

## Methods

### Search strategy

The conduct of the systematic review adhered to the Preferred Reporting Items for Systematic Reviews and Meta-Analyses (PRISMA) guidelines (http://www.prisma-statement.org/) [27]. The PRISMA Statement has emerged as the best tool to help authors improve the reporting of systematic reviews and meta-analyses. Based on these guidelines, a comprehensive search of the following computerized bibliographic databases was conducted from April to May 2012: MEDLINE with Full Text, CINAHL with Full Text, and PsycINFO (EBSCOhost) as well as Embase (Ovid) covering the period from January 1970 to April 2012. Using relevant Mesh words or sub-headings, the following combination of key words were used for our search:

(Questionnaire* OR tool* OR instrument* OR scale* OR survey*)

and

(urbanicity OR urbanisation OR urbanization)

The bibliographical references of retrieved articles and previous available literature reviews on the relationship between urbanisation and health were manually searched, complemented by a citation tracking of articles using the Web of Science databases and Google scholar, to identify additional citations.

### Inclusion and exclusion criteria

Studies included in this systematic review were those that focused on the development of an urbanicity scale with clearly defined items or the adoption of an existing scale, included at least one outcome measure related to health, were published in peer-reviewed journals, the full text was available in English, and tested for validity and reliability. Validity was assessed by considering the urbanicity scale’s content validity, that is, whether the items of the urbanicity scale were generated following explicit a priori theoretical framework or knowledge [3], and construct validity, that is, whether the urbanicity scale had valid content in terms of the content being positively related to urbanicity, was associated with a health outcome or risk factors, and was psychometrically derived (factorial evidence) [28]. Reliability was assessed by considering the scale’s internal consistency (assessed by the Cronbach’s alpha coefficient, representing correlations between urbanicity scale’s items to ascertain that they produce similar scores) and test–retest reliability (assessed by the intraclass correlation coefficients to establish the consistency and stability of the urbanicity scale from one time to another) [3]. Studies were excluded that used surrogate or generic measures of urbanisation such as the rural/urban dichotomy, population density, or mathematical modelling [29] and were published in a language other than English. Theses, books and non-peer reviewed materials were also excluded.

### Data extraction and quality assessment

**Figure 1:**
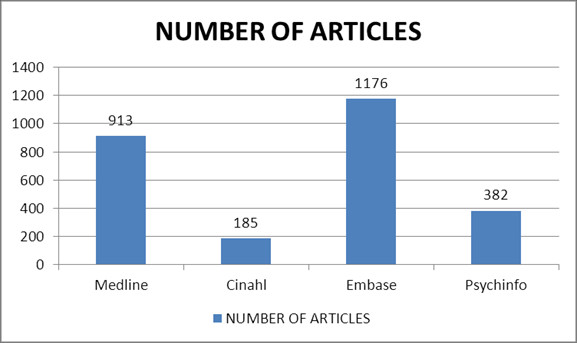
Summary of database search (Jan 1970-April 2012).

Data were extracted according to the PRISMA guidelines [27] using the Outcome Measures Rating Form Guidelines [30,31] The Outcome Measures Rating Form Guidelines permits the assessment and evaluation of the properties (validity and reliability) as well as clinical utility of outcome measures [31]. All searches were stored using an EndNote library. Using this library, all potentially relevant studies were screened by one of the reviewers (SC) as part of the preliminary inclusion/exclusion using the title as well as reading the abstract of each retrieved article. In the final inclusion/exclusion phase, papers retained for inclusion in the preliminary phase were independently reviewed by two reviewers (SC and JO) (Figure 1). The review extracted data related to the development of scales and whether the development of the scale was informed by a theoretical framework, their validity and reliability, as well as demographic characteristics (e.g. year of publication, journal of publication, country of origin, sample size, any health outcome measures, number of items of the scale and method of data collection). Any disagreements over which paper to include/exclude or over the extracted data were discussed with a third reviewer (AR) until a final consensus reached between the three reviewers. Because the scale properties can be influenced by many factors such as the sample size or the analytical approach adopted, poor-quality studies were classified as those with inadequate sample size, poor definition or conceptualisation of urbanicity, and unclear analytical frameworks or hypotheses being tested (not informed by any theoretical framework) [32]. However good quality studies were those where an underlying theory guiding scale construction was clearly stated, content validation of scale items was described, internal consistency was reported to be above 0.70, a priori hypotheses were confirmed, and the scale was supported by a dimensional structure through either exploratory or confirmatory factor analyses [33].

### Data analysis

The retained studies were heterogeneous in terms of study design and measurement of urbanicity, hence the analysis focused on the narrative, absolute and relative frequencies, and contingency tables of ratings [33].

## Results

**Figure 2:**
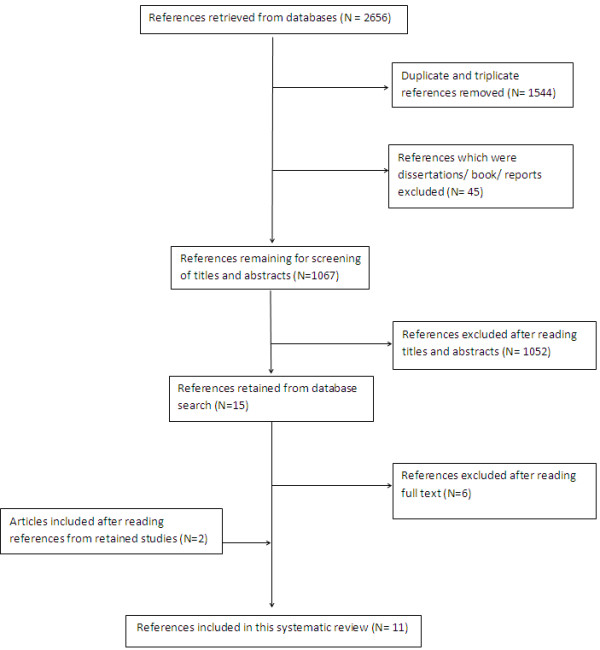
Flow chart of study selection.

We identified 2656 relevant abstracts of which 1589 were immediately excluded because they did not meet inclusion criteria (n = 1544 were duplicates; n = 45 books/reports). The remaining abstracts were read and a further 1052 excluded because they were not relevant. The full text of the remaining 15 articles was read and 6 were excluded because they did not meet the inclusion criteria. After hand searching related reference lists 2 more studies were added leaving 11 included studies (Figure 2).

### Summary of included studies

A summary of the included studies including the study characteristics, the number of scale items, and whether reliability and validity properties were discussed are presented in Table 1. Studies were conducted in the Sri Lanka, Austria, China, Nigeria, India and Philippines and published between 2001 and 2012. They ranged in size from 3327 to 33,404 participants. The number of scale items ranged from 7 to 12 items in 5 studies [3,8,19,23,34] and was not presented in 5 studies [4,17,20,35,36]. One study measured urban area socioeconomic disadvantage instead of urbanicity [37].

**Table 1:** Characteristic of the scales used in the review

**Study, author, country**	**N**	**Study characteristics**	**No. of scale items**	**Methods of data collection**	**Reliability and validityproperties discussed Yes/No**	**Duration of scale**
Allender et al. 2011, Sri Lanka [8]	4485	>18 yrs male and female	10	Interviewer –administered questionnaire for individual level and personal/telephone interview for village heads for community level	No	Not given
Vavken et al. 2011, Austria [35]	14,507	Mean age 36 yrs	Not given	Survey	No	Not given
	6569 men	45% male				
		55% female				
	7938 women					
Jones-Smith et al. 2010, China [34]	218 provinces in China		12	Individual, household and community level surveys	Yes	Data from survey used to construct the scale
Antai et al. 2010, Nigeria [37]	Children born to 2118 mothers	Children under 5 yrs	Scale measured urban area disadvantage and not urbanicity	Data from 2003 Nigeria Demographic and Health Survey used	No	Not given
Monda et al. 2007, China [23]	8760	Men and women aged between 18 and 55	10 items	Data derived from CHNS survey	No	Not given
Van de Poel et al. 2009, China [17]	6484	>16 years	Not mentioned	Individual and community surveys	Yes	no
Allender et al. 2010, India [19]	3705	Men and women aged 15 – 64 years	7	Individual and household surveys	Only face validity discussed	Not given
Dahly et al. 2007, Phillipines [3]	3327	Any woman giving birth between May 1 1983 to April 30 1984	7	Individual and community surveys	Yes	Not given
McDade et al. 2001, Phillipines [4]	3327	Pregnant women	Not mentioned	Individual, household and community surveys	No	no
Liu et al. 2003, China [36]	33,404 individuals	Mean age 28.9 years	Not mentioned	Individual and household surveys	No	no
Van de Poel et al. 2012, China [20]	31,333 person-wave observations across 5 waves	Not specified	Adapted from Van de Poel 2009	Individual and community surveys (with community heads)	No	Not given

### The methods adopted in the development of the scales in included studies

A variety of methods were used in scale development in the studies and included literature review [3,34], empirical investigations [8,19,23,34], and expert panels [34] (Table 2). Content validity was assessed in two studies [3,34], reliability was assessed in four studies [3,17,19,34] construct validity in four studies [3,4,17,34]. Reliability was most commonly assessed by measures of internal consistency in % of studies [3,17,19,34]. Construct validity was most commonly assessed by exploratory factor analysis in 27% of studies [4,17,34].

**Table 2:** Methods adopted in the development of the scales included in the review

**Study**	Item development*			**Content validity**			**Reliability+**			**Construct validity±**	**Exploratory factor analysis**	**Criterion validity**
	**Literature review**	**Empirical study**	**Panel of experts**	**Review by target population. through, pre-tests, pilot studies**	**Panel of experts**	**Literature review**	**Internal consistency**	**Test-retest reliability**	**Item/ total correlation**			
Allender et al. 2011 [8]	✗	✓	✗	✗	✗	✗	✗	✗	✗	✗	✗	✗
Vavken et al. 2011 [37]	✗	✗	✗	✗	✗	✗	✗	✗	✗	✗	✗	✗
JC Jones-Smith et al. 2010 [34]	✓	✓	✓	✓	✓	✓	✓	✓	✓	✓	✓	✓
Antai et al. 2010 [39]	✗	✗	✗	✗	✗	✗	✗	✗	✗	✗	✗	✗
Monda et al. 2007 [23]	✗	✓	✗	✗	✗	✗	✗	✗	✗	✗	✗	✗
Van de Poel et al. 2012 [20]	✗	✗	✗	✗	✗	✗	✗	✗	✗	✗	✗	✗
Allender et al. 2010 [19]	✗	✓	✗	✗	✗	✗	✓	✗	✗	✗	✗	✗
Dahly et al. 2007 [3]	✓	✗	✗	✗	✗	✓	✓	✓	✓	✓	✗	✓
McDade et al. 2001 [4]	✗	✗	✗	✗	✗	✗	✗	✗	✗	✗	✓	✗
Liu et al. 2003 [36]	✗	✗	✗	✗	✗	✗	✗	✗	✗	✗	✗	✗
Van de Poel et al. 2009 [17]	✗	✗	✗	✗	✗	✗	✓	✓	✗	✓	✓	✓

*No studies used focus groups, interview/key informant interview or authors personal preferences during item development; + no studies used split half reliability or inter-observer reliability during reliability assessment; ± no studies tested convergent validity or used principal component analysis of extreme group comparisons, confirmatory factor analysis, discriminant analysis or structural equation modelling to test construct validity.

### Quality ratings for each of the scales in included studies

One study followed an explicit a priori theoretical framework [3]. Three studies reported on content validity as well as reported high reliability scores (i.e. above 0.70) [3,19,34]. All studies confirmed at least 75% of hypotheses relating to the constructs under consideration. One study was rated as high quality (rating score >4 out of 5) (Table 3) [34].

**Table 3:** Ratings for each of the scales included in the review

**Scale name**	Quality score* (out of 5)	**Followed an a priori explicit theoretical framework**	**Reported efforts towards content validation**	**Reliability scores above 0.7**	**At least 75% of the Hypotheses regarding relation-ships with the construct under consideration were confirmed?**	**Conceptual dimensional structure was supported by means of factor analysis?**
Adapted scale from Dahly and Adair 2007 (Allender 2011) [8]	2 - poor	+	-	-	+	-
Adopted the NUTS framework to measure urbanisation (Vavken et al. 2011) [35]	1 - poor	-	-	-	+	-
Urbanicity scale (Jones et al. 2010) [34]	4 - high	-	+	+	+	+
Urbanicity scale developed by Mendes and Popkin 2005 (Antai et al. 2010) [37]	1 - poor	-	-	-	+	-
Urbanicity index (Van de Poel 2012) [20]	2 - poor	-	-	-	+	+
Adaptation of Dahly and Adair scale (Allender et al. 2010) [19]	3 - medium	-	+	+	+	-
Multi-component urbanicity scale for Metro Cebu (Dahly and Adair2007) [3]	3 - medium	-	+	+	+	-
Factor analysis as a tool to measure urbanization (McDade and Adair 2001) [4]	2 - poor	-	-	-	+	+
Urbanization index (Liu et al. 2003) [36]	1 - poor	-	-	-	+	-
Urbanicity index (Van de Poel 2009) [17]	2 - poor	-	-	-	+	+

– not assessed + assessed and positive result; *Quality Score calculated by assigning 1 point for each criteria listed as present (‘+’); Quality ranking: ≤2 = poor quality; 3 = medium quality; ≥ 4 = high quality.

### Summary of the studies included and the psychometric properties of scales used

Of the included studies one was a cohort study [8], four were cross-sectional studies [19,34,35,37] and six were secondary analyses of data [3,4,17,20,23,36]. One was conducted in a developed country [35] the rest in low to middle income countries. All studies reported an association of urbanicity or urbanisation and health outcomes except one [4]. Four of eleven included studies used the terms urbanisation and urbanicity interchangeably [8,17,19,20]. Three of the included studies reported on the validity and reliability of the instruments used [3,19,34] and only one study was assessed to be high quality [34].

Allender et al. [8] conducted a cohort study which aimed to evaluate the extent to which urbanisation was a risk factor for self-reported non-communicable diseases. The study was conducted in a representative sample from seven of the nine provinces in Sri Lanka (n = 4,485; response rate = 89.7%; >18 years of age). The authors developed a 7-item urbanicity scale from urban characteristics such as population size, population density, and access to markets, transportation, communications/media, economic factors, environment/sanitation, health, education, and housing quality. They assigned a maximum of 10 points to each item of the urbanicity scale resulting in score from 0 (no urbanicity) to 70 (high urbanicity). The village administrators in 100 study villages provided the relevant information for their village. These scores were grouped into tertiles of urbanicity (1 low urbanicity, 2 medium and 3 high urbanicity) for subsequent analysis. The authors found that urbanicity was positively associated with physical inactivity, high body mass index and diabetes mellitus in men and women. However, the validity and reliability of the urbanicity scale were not reported.

Vavken et al. [35] conducted a cross-sectional study in 14,507 men and women in Austria (mean age 36 years; 45% male) which aimed to determine the burden of musculoskeletal disease by urbanicity, socioeconomic status, age and sex. They adopted the Nomenclature of Territorial Units for Statistics III classification of urbanicity ranging for 1 (rural areas) to 3 (urban areas) to measure urbanisation. The Nomenclature of Units for Territorial Statistics (NUTS) is a geocode standard for referencing the subdivisions of countries within the European Union for statistical purposes [38]. The authors referenced the NUTS website but they did not present further detail about the validity and reliability characteristics of the NUTS scale in the paper. They found strong evidence for an association between urbanicity and arthritis and osteoporosis but not spinal conditions.

Jones-Smith et al. [34] conducted a cross-sectional study in China which aimed to develop an urbanicity scale from existing data, test whether the scale was reliable and valid, and assess whether it provided information beyond what could be determined from the traditional urban/rural dichotomous variables. They used the procedures for building scales developed by DeVellis 2003 [39] and Netemeyer, Beardon and Sharma 2003 [40] to construct their scale. This involved first, consulting authoritative sources such as previous literature and content experts to establish a strong definition of the construct they intended to measure. Second, they identified which variables were available to represent those defining concepts as well as how each should be scored. Third, they tested the scales performance as a measurement tools including its uni-dimensionality, reliability, content, criterion and construct validity. They identified 12 main components thought to define urbanicity which were: population density, economic activity traditional markets, modern markets, transportation infrastructure, sanitation, communications, housing, education, diversity, health infrastructure, social services. They allocated a maximum of 10 points each to each of the 12 components. The 12 components appeared to represent a unidimensional underling construct (called urbanicity) as evidenced by high eigenvalue of only one factor in the exploratory factor analysis. The scale had good internal consistency (Cronbach alpha values = 0.85 to 0.89). The scale exhibited temporal stability in test- retest evaluations (correlations r = 0.90 to 0.94). There was some evidence for criterion validity from its comparison to the official classification of communities as urban or rural (Kappa statistic for agreement beyond chance of their scale with the “gold standard” = 0.21 to 0.48). Linear and logistic regression indicated that their scale demonstrated good construct validity: increasing scores on the urban scale were significantly associated with increases in the adjusted per capita household income and with significantly lower odds of having more than one child. They demonstrated that the scale predicted the incidence of overweight/obesity populations in China and added valuable additional information compared to the traditional measure namely the urban–rural dichotomy.

Antai et al. [37] conducted a cross-sectional study in Nigeria among 2118 children aged less than five years, which assessed whether urban area socioeconomic disadvantage has an impact on under-five mortality. Urban under-five mortality rates were directly estimated from the 1990, 1999, and 2003 Nigeria Demographic and Health Surveys. Urban area disadvantage was measured using the urban area disadvantage index (UADI) score. The UADI scores reflect the overall level of urban area disadvantage based on eight indicators of socioeconomic disadvantage at the neighbourhood level. The UDAI scores were generated through principal component analysis using 165 out of 365 available primary sampling units (PSUs). The PSUs were administratively defined, homogenous areas used as proxies for ‘neighbourhoods’ or ‘communities’ consisting of a minimum of 50 households per PSU. The scale measured ‘urban area disadvantage’ (e.g. children living in a household without piped water, flush toilets or electricity and other amenities) rather than urbanicity (i.e.urban conditions at any given point in time). The authors found that urban area disadvantage was significantly associated with under-5 mortality after adjustment for individual child and mother level demographic and social characteristics.

Monda et al. [23] used an existing longitudinal dataset from the China Health and Nutrition Survey (CNHS) (wave from 1991–1997; n = 8769, male = 50%; 18–55 years of age) to examine the effect of rapid urbanisation on the occupational physical activity patterns of Chinese adults. The authors utilized a multidimensional measure designed specifically for the CHNS to capture urbanisation from the physical, social, cultural and economic environments. The urbanisation variable was developed using data from community surveys and household-level information and comprised 10 components which were: communication, economic, housing-related and transportation infrastructure, the availability of schools, markets and health care environmental sanitation and population size and density. The data were used to generate a continuous variable called an ‘urbanisation score’ for each community for each data collection period where each component was assigned 10 possible points and summed for a maximum value of 100 points (100 = high urbanisation). The properties of the scale were not further commented on in the paper although readers were referred to a reference (under review) in which further details on the development of the urbanicity index could be found. They found that men had 68% greater odds, and women had 51% greater odds, of light versus heavy occupational activity given the mean change in urbanisation over the 6-year period. Further, simulations showed that light occupational activity increased linearly with increasing urbanisation. After controlling for individual-level predictors, community-level urbanisation explained 54% and 40% of the variance in occupational activity for men and women, respectively. The authors concluded that because occupational activity remains the major source of energy expenditure for adults, the Chinese population is at risk of dramatic increases in the numbers of overweight and obese individuals.

Van de Poel et al. [17] also used longitudinal data from the CNHS (6484 adults aged >16 years and older) to investigate the role of urbanisation and the spread of non-communicable diseases in China. They developed an urbanicity index by firstly applying factor analysis to a set of 25 community level characteristics (e.g.number of bus stations, dirt roads, primary schools) that reflect a community’s level of urbanicity. They subsequently computed a rank-based measure of inequality in disease risk factors by degree of urbanicity. The first factor was retained as it explained the highest proportion of the common variance among community variables (~47%). Factor loadings were then computed which were the degree to which the remaining characteristics correlated with the first factor and range for -1 to +1. The urbanicity index was then constructed as a linear combination of all these community characteristics weighted by their factor loading using an oblique promax rotation. The authors report that the index had internal consistency, temporal stability, criteria-related validity and construct validity. The authors conclude that their urbanicity index appears to be a plausible indicator of the degree of urbanicity of communities in China. In relation to non-communicable diseases, at the individual level low engagement in physical activity and farming explain more than half of the urban concentration of overweight and a rising share (28%) of the greater prevalence of hypertension in more urbanised areas.

Allender et al. [20] conducted an investigation into the association of urbanization and non-communicable disease risk factors in 2705 men and women aged 15–64 years in Tamil Nadu, India. They adopted a modified version of a composite continuous measures of urbanicity previously used and validated for the Philippines. It comprised seven elements: population size, population density, access to markets, communications, transport, education and health services. The authors modified it by using only three variables: population size, population density, and education. They assigned a maximum of 10 points to each item to generate a modified scale (range 0 (no urbanicity) to 70 (high urbanicity). They conducted validity testing on the modified scale and obtained a Cronbach alpha reliability coefficient of 0.72. Using this scale urbanicity appeared to be consistent with exiting government definitions or ‘urban’ and ‘rural’. Face validity was discussed by the authors but no other scale properties. The scale was used in conjunction with data collected from 3705 participants in the World Health Organization’s 2003 STEPwise risk factor surveillance survey in Tamil Nadu, India. Linear and logistic regression were used to examine the relationship between urbanicity using this scale and chronic disease risk. Using the urbanicity index the authors found that increased urbanicity was positively associated with body mass index, low physical activity and mean number of servings of fruit and vegetables consumed per day (P < 0.05) in men and women.

Dahly et al. [3] conducted a study in which they aimed to construct a scale of urbanicity using community level data from the Cebu Longitudinal Health and Nutrition Survey (CNHLS) in the Phillipines [41]. They used the scale development method of De Velliss 2003 [39] to validate the new measure and tested its performance against the urban–rural dichotomy. Items included in the urbanicity scale were population size, population density, communications, transportations, education facilities, health services and markets. Each item was scored 0 to 10 scale so the scale ranged from 0 (no urbanicity) to 70 (high urbanicity). The scale had high internal consistency (item spearman correlations r > 0.5, P < 0.001; Cronbach alpha range 0.87 to 0.89), high temporal stability (spearman correlations r = 0.85 to 0.97, P < 0.001) high content validity, criterion validity and construct validity. The new scale illustrated misclassification by the urban–rural dichotomy, and was able to detect differences in urbanicity, both between communities and across time, that were not apparent before. The authors concluded that the new scale was a better measure of urbanicity than the traditionally used urban–rural dichotomy. For example, in generalised linear models applied to the CNHLS data, the scale was found to explain the variation in calorie intake above and beyond that explained by the urban–rural dichotomy alone.

McDade et al. [4] conducted an analysis of multiple definitions of urbanicity also using data from the Cebu Longitudinal and Health Nutrition Survey in the Philippines [41]. Factor analysis was conducted on 27 household and 26 community variables using principle components analysis. This generated factor scores that summarised a household’s position with respect to access to infrastructure and health services and level of affluence. Extensive comparisons of factor scores were then made across urban and rural areas, and across settlement types to explore household and community level markers of urbanicity differentiating households in geographically defined urban and rural areas. High population density, the availability of telephone, mail, transportation services, electricity, clean water and health care facilities were found to be the correlates of urbanicity. Apart from factor analysis, the results of further reliability and validity property evaluations were not reported in this paper. The relationship between urbanicity and health outcomes was also not reported in this paper.

Liu et al. [36] used the CHNS to investigate the effects of urbanisation on health care and health insurance in rural China among 33,404 men and women (mean age 28.9 years) using individual and community surveys. The authors used three sets of variables to construct an urbanisation index: 1) total population of the neighbourhood divided by the area of the neighbourhood; 2) infrastructure variables 3) industrialisation variables. The authors first calculated the distribution of these variables and then defined the uppermost quartile as a high level, the lowermost quartile as the low level and the middle two quartiles as the middle level. They did not describe the psychometric characteristics of the urbanisation index. The primary finding was that urbanisation leads to a significant and equitable increase in insurance coverage, which in turn plays a critical role in access to health care.

Van de Poel et al. [20] investigated adverse health effects of rapid urbanisation in China using the CHNS panel data for 1991–2004. The authors constructed an urbanicity index using factor analysis on a broad set of characteristics from the CHNS community level data pooled across all survey sites as described previously [17]. The urbanicity index captured information on population size, land use, transportation facilities, economic activity and public services. The validity of the urbanicity index has previously been reported [17] and was not described in the paper. The index correlates with a subjective classification of communities as urban, suburb, town or rural. The authors found that greater urbanisation increased the likelihood of reporting of poor health.

## Discussion

The purpose of this study was to assess the reliability and validity of the available urbanicity scales and identify areas where more research is needed to facilitate the development of a standardised measure of urbanicity. After a thorough search of the literature we found eleven relevant studies. Our main finding is that in eight of eleven included studies the properties of the instruments were not reported. Only one paper used an a priori theoretical framework in the use of their urbanicity scale. Three (27%) tested content validity and the same three tested reliability. Despite this, several studies reported significant associations between the measure of urbanicity and dependent variables. Although we found several studies which investigated associations between urbanicity and health, the majority (73%) of included studies did not report the properties of the urbanicity scales. Therefore, the properties of urbanicity scales have not been comprehensively established. Our findings suggest that the development, testing and standardisation of an international urbanicity scale is urgently needed and the association between urbanicity and health re-assessed using a tested, valid, and reliable urbanicity scale.

Studies that did report on the properties of the urbanicity index found that increasing urbanicity had predominantly negative consequences on health, for example, resulting in increased body mass index, low physical activity and reduced mean number of servings of fruit and vegetables consumption [19]. There was evidence of some positive consequence of urbanicity namely significant and equitable increases in insurance coverage [36]. Interestingly, four of eleven included studies used the terms urbanisation and urbanicity interchangeably [8,17,19,20] failing to differentiate two key dimensions namely urban living as a dynamic process changing over time (urbanisation) and prevalent urban characteristics i.e. characteristics at a given point in time (urbanicity). More research is needed to examine the context of urbanisation and urbanicity by economic development (e.g. developed and developing countries).

Some studies examined the relationship between urbanisation and risk factors for self-reported non-communicable diseases in Sri Lanka [8] and India [19] and non-communicable diseases in China [17,20] using an urbanicity scale. Although urbanisation and urbanicity are interrelated they are different. For example, the prevalence of low socioeconomic status groups (a measure of urbanicity) will have an impact on the overall health of the city’s population (as poverty is associated with poorer health outcomes). However, the rate of economic investment (a measure of urbanisation) will affect the socioeconomic status of a city which will in turn affect the health status of the city’s population. It is possible that increased urbanisation is associated with greater utilisation of health services. Future research on the association between urbanicity and health outcomes needs better clarity and consistency in the use of measures of urbanicity versus urbanisation.

### Research agenda

Future multidisciplinary studies are needed to clearly differentiate the two key elements of urban environments, urbanicity and urbanisation. There is an urgent need of studies to develop and standardise measures of urbanicity. Within and between city studies are needed to help identify features of the urban environment that are associated with poor health and other features that may be salutogenic. Finally, longitudinal cohort studies to confirm the relationship between increased urbanicity and health outcomes are urgently needed.

## Conclusion

Given the cross-sectional nature of the included studies, the relationship between increased urbanisation and health outcomes cannot be used to establish causality. Furthermore, the studies included were those published in English because we did not have enough financial and logistical structure (e.g. translation services) to include studies published in languages other than English. This could limit the external validity of the findings reported in this study. Notwithstanding these limitations, the emerging evidence is that increased urbanisation is associated with deleterious health outcomes. It is possible that increased urbanisation is also associated with access and utilisation of health services. However, urbanicity measures differed across studies, and the properties of the used scales were not developed in the majority of studies.

## Competing interests

The authors declare that we have no competing interests.

## Authors’ contributions

SC conducted the literature searches, screened all potentially relevant studies as part of the preliminary inclusion/exclusion and reviewed papers retained for inclusion. JO reviewed papers retained for inclusion and wrote the results and discussion section. AR conceived and designed the study and wrote the introduction and methodology sections. AR also reviewed papers for which there was disagreement as to whether they should be included. All authors reviewed drafts of the manuscript and approved the final version.

## Pre-publication history

The pre-publication history for this paper can be accessed here:

http://www.biomedcentral.com/1471-2458/13/513/prepub

## References

[B1] United Nations World Urbanization Prospects: The 2007 Revision Population. ESA/P/WP/205 In 2008 New York: Population Division, Department of Economic and Social Affairs, United Nations

[B2] Allender S Foster C Hutchinson L Arambepola C Quantification of urbanization in relation to chronic diseases in developing countries: a systematic review J Urban Health 2008 85 6 938 951 10.1007/s11524-008-9325-4 18931915 PMC2587653

[B3] Dahly DL Adair LS Quantifying the urban environment: a scale measure of urbanicity outperforms the urban–rural dichotomy Soc Sci Med 2007 64 7 1407 1419 10.1016/j.socscimed.2006.11.019 17196724 PMC2001275

[B4] McDade TW Adair LS Defining the “urban” in urbanization and health: a factor analysis approach Social Science &amp; Medicine 2001 53 1 55 70 10.1016/S0277-9536(00)00313-0 11380161

[B5] Vlahov D Galea S Urbanization, urbanicity, and health J Urban Health 2002 79 4 Suppl 1 S1 S12 12473694 10.1093/jurban/79.suppl_1.S1 PMC3456615

[B6] Bradshaw YW Urbanization and underdevelopment: a global study of modernization, urban bias, and economic dependency Am Sociol Rev 1987 52 2 224 239 10.2307/2095451

[B7] Wikström P-OH Dolmén L Urbanisation, neighbourhood social integration, informal social control, minor social disorder, victimisation and fear of crime International Review of Victimology 2001 8 2 121 140 10.1177/026975800100800202

[B8] Allender S Wickramasinghe K Goldacre M Matthews D Katulanda P Quantifying urbanization as a risk factor for noncommunicable disease J Urban Health 2011 88 5 906 918 10.1007/s11524-011-9586-1 21638117 PMC3191205

[B9] Sobngwi E Mbanya J-C Unwin NC Porcher R Kengne A-P Fezeu L Minkoulou EM Tournoux C Gautier J-F Aspray TJ Exposure over the life course to an urban environment and its relation with obesity, diabetes, and hypertension in rural and urban Cameroon Int J Epidemiol 2004 33 4 769 776 10.1093/ije/dyh044 15166209

[B10] Weng X Liu Y Ma J Wang W Yang G Caballero B An urban–rural comparison of the prevalence of the metabolic syndrome in Eastern China Public Health Nutr 2007 10 2 131 136 17261221 10.1017/S1368980007226023

[B11] Ramachandran A Mary S Yamuna A Murugesan N Snehalatha C High prevalence of diabetes and cardiovascular risk factors associated with urbanization in India Diabetes Care 2008 31 5 893 898 10.2337/dc07-1207 18310309

[B12] Passchier-Vermeer W Passchier WF Passchier-Vermeer W Passchier WF Noise exposure and public health Environmental Health Perspectives 2000 108 Suppl 1 123 131 Date of Publication: 2000; 200 10698728 10.1289/ehp.00108s1123 PMC1637786

[B13] Gehring U Wijga AH Brauer M Fischer P de Jongste JC Kerkhof M Oldenwening M Smit HA Brunekreef B Traffic-related Air pollution and the development of asthma and allergies during the first 8 years of life Am J Respir Crit Care Med 2010 181 6 596 603 10.1164/rccm.200906-0858OC 19965811

[B14] Lin RS Sung FC Huang SL Gou YL Ko YC Gou HW Shaw CK Role of urbanization and air pollution in adolescent asthma: a mass screening in Taiwan J Formos Med Assoc 2001 100 10 649 655 11760369

[B15] Broms K Norback D Eriksson M Sundelin C Svardsudd K Effect of degree of urbanisation on age and sex-specific asthma prevalence in Swedish preschool children BMC Publ Health 2009 9 303 10.1186/1471-2458-9-303 PMC2741449 19695101

[B16] Popkin BM The nutrition transition and its health implications in lower-income countries Public Health Nutr 1998 1 1 5 21 10555527 10.1079/phn19980004

[B17] Van de Poel E O'Donnell O Van Doorslaer E Urbanization and the spread of diseases of affluence in China Econ Hum Biol 2009 7 2 200 216 10.1016/j.ehb.2009.05.004 19560989

[B18] Miranda JJ Gilman RH Smeeth L Differences in cardiovascular risk factors in rural, urban and rural-to-urban migrants in Peru Heart 2011 97 10 787 796 10.1136/hrt.2010.218537 21478383 PMC3183994

[B19] Allender S Lacey B Webster P Rayner M Deepa M Scarborough P Arambepola C Datta M Mohan V Level of urbanization and noncommunicable disease risk factors in Tamil Nadu, India Bull World Health Organ 2010 88 4 297 304 10.2471/BLT.09.065847 20431794 PMC2855597

[B20] Van De Poel E O'Donnell O Van Doorslaer E Is there a health penalty of China’s rapid urbanization? Health Econ 2012 21 4 367 385 10.1002/hec.1717 21341344

[B21] Carbajal-Arroyo L Barraza-Villarreal A Durand-Pardo R Moreno-Macias H Espinoza-Lain R Chiarella-Ortigosa P Romieu I Impact of traffic flow on the asthma prevalence among school children in Lima Peru. Journal of Asthma 2007 44 3 197 202 10.1080/02770900701209756 17454338

[B22] Gul H Gaga EO Dogeroglu T Ozden O Ayvaz O Ozel S Gungor G Respiratory health symptoms among students exposed to different levels of air pollution in a Turkish city International Journal of Environmental Research & Public Health [Electronic Resource] 2011 8 4 1110 1125 21695031 10.3390/ijerph8041110 PMC3118880

[B23] Monda KL Gordon-Larsen P Stevens J Popkin BM China's transition: the effect of rapid urbanization on adult occupational physical activity Soc Sci Med 2007 64 4 858 870 10.1016/j.socscimed.2006.10.019 17125897 PMC2753984

[B24] Greif MJ Dodoo FN-A Jayaraman A Urbanisation, poverty and sexual behaviour: the tale of five African cities Urban Studies 2011 48 5 947 957 10.1177/0042098010368575 21744541

[B25] Leviton LC Snell E McGinnis M Urban issues in health promotion strategies Am J Public Health 90 6 863 866 10846502 10.2105/ajph.90.6.863 PMC1446271

[B26] Fujiwara T Takano T Nakamura K The spread of drug abuse in rapidly urbanizing communities in Vientiane, Lao People's Democratic Republic Health Promot Internation 2005 20 1 61 68 10.1093/heapro/dah508 15668213

[B27] Moher D Liberati A Tetzlaff J Altman DG Preferred reporting items for systematic reviews and meta-analyses: the PRISMA statement Ann Intern Med 2009 151 4 264 269 10.7326/0003-4819-151-4-200908180-00135 19622511

[B28] Kaplan RM Bush JW Berry CC Health status: types of validity and the index of well-being Health Serv Res 1976 11 4 478 507 1030700 PMC1071947

[B29] Bettencourt LM Lobo J Strumsky D West GB Urban scaling and its deviations: Revealing the structure of wealth, innovation and crime across cities PloS One 2010 5 11 e13541 10.1371/journal.pone.0013541 21085659 PMC2978092

[B30] Butt TA McCarl BA Angerer J Dyke PT Stuth JW The economic and food security implications of climate change in Mali CliM Chang 2005 68 3 355 378 10.1007/s10584-005-6014-0

[B31] Glewwe P Miguel EA Schultz TP, Strauss JA The impact of child health and nutrition on education in less developed countries Handbook of Development Economics. Volume 4 2007 Amsterdam: North Holland Press 3561 3606

[B32] Egger M Smith GD Altman DG Systematic reviews in health care. Meta-analysis in context 2001 London: BMJ Books

[B33] Jamnadass R Dawson I Franzel S Leakey R Mithöfer D Akinnifesi F Tchoundjeu Z Improving livelihoods and nutrition in sub-Saharan Africa through the promotion of indigenous and exotic fruit production in smallholders' agroforestry systems: a review Int For Rev 2011 13 3 338 354

[B34] Jones-Smith JC Popkin BM Understanding community context and adult health changes in China: development of an urbanicity scale Soc Sci Med 2010 71 8 1436 1446 10.1016/j.socscimed.2010.07.027 20810197 PMC2942954

[B35] Vavken P Dorotka R Burden of musculoskeletal disease and its determination by urbanicity, socioeconomic status, age, and sex: results from 14,507 subjects Arthritis Care Res 2011 63 11 1558 1564 10.1002/acr.20558 21793230

[B36] Liu GG Wu X Peng C Fu AZ Urbanization and health care in rural China Contemp Econ Policy 2003 21 1 11 24 10.1093/cep/21.1.11

[B37] Antai D Moradi T Urban area disadvantage and under-5 mortality in Nigeria: The effect of rapid urbanization Environ Health Perspect 2010 118 6 877 883 10.1289/ehp.0901306 20146963 PMC2898867

[B38] Commission E Eurostat Introductionhttp://epp.eurostat.ec.europa.eu/portal/page/portal/nuts_nomenclature/introduction. Acceessed 10 March 2013

[B39] DeVellis RF Scale Development: Theory and Applications 2003 Second Applied Social Research Methods

[B40] Netemeyer R Beardon W Sharma S Scaling procedures: issues and applications 2003 Thousand Oaks, California: Sage Publications

[B41] Cebu Study Team Underlying and proximate determinants of child health: the Cebu longitudinal health and nutrition study Am J Epidemiol 1991 133 2 185 201 1985447

